# Integrative SMR and single cell & spatial analysis reveals the spatial heterogeneity and prognostic value of CASP9-mediated apoptotic pathways in clear cell renal cell carcinoma

**DOI:** 10.1007/s12672-025-03798-0

**Published:** 2025-11-07

**Authors:** Zijie Yu, Xinghan Yan, Wenchuan Shao, Lingchen Cai, Da Zhong, Xiyi Wei, Ninghong Song

**Affiliations:** 1 Department of Urology, The First Affiliated Hospital of Nanjing Medical University, Nanjing, 210029 China; 2 Department of Urology, The Affiliated Cancer Hospital of Nanjing Medical University & Jiangsu Cancer Hospital & Jiangsu Institute of Cancer Research, Nanjing, 210029 China

**Keywords:** CcRCC, CASP9, Apoptosis, Single-cell RNA sequencing, Spatial transcriptomics, SMR, Prognostic model

## Abstract

**Background:**

Clear cell renal cell carcinoma (ccRCC) is the most common histological subtype of renal cancer and remains a clinical challenge due to its frequent resistance to therapy and poor prognosis in advanced stages. Apoptosis, a fundamental tumor-suppressive mechanism, exhibits paradoxical roles in cancer, wherein apoptotic tumor cells can also contribute to immunosuppression and tumor progression. However, the spatial dynamics, transcriptional heterogeneity, and prognostic relevance of apoptosis-related gene programs in ccRCC remain poorly defined.

**Methods:**

We performed an integrative analysis combining single-cell RNA sequencing (scRNA-seq), spatial transcriptomics, and summary-based Mendelian randomization (SMR) to dissect apoptosis-related malignant cell states in ccRCC. Cancer cells were stratified based on apoptosis gene signatures and *CASP9* expression. Cell–cell communication was assessed using CellChat and spatial interaction networks were constructed using RCTD and mistyR. SMR was employed to link genetically regulated *CASP9* expression with renal cancer risk. A *CASP9*-associated prognostic model was developed using LASSO Cox regression and DeepSurv on TCGA and E-MTAB-1980 cohorts.

**Results:**

We identified transcriptionally and spatially distinct apoptosis-high and apoptosis-low malignant cell subpopulations. Apoptosis-high tumor cells, characterized by elevated *CASP9* expression, preferentially localized near macrophage-enriched stromal regions and exhibited stronger spatial clustering. Ligand–receptor modeling revealed directional signaling via the SPP1–CD44 axis between *CASP9*-high cancer cells and macrophages. SMR analysis provided genetic evidence supporting *CASP9* as a causal gene for renal cancer. *CASP9*-high cells demonstrated distinct developmental trajectories and formed multicellular spatial modules with macrophages and cycling cells. A five-gene apoptosis-related signature derived from *CASP9*-stratified tumor cells robustly predicted patient survival across both training and validation cohorts. Low-risk patients exhibited enriched immune infiltration, increased immune checkpoint expression, and enhanced immune pathway activity.

**Conclusions:**

Our study reveals that apoptosis, particularly *CASP9*-driven programs, defines a spatially organized, immunosuppressive malignant cell state in ccRCC. *CASP9* acts as both a genetic driver and spatial regulator of tumor–macrophage interactions, contributing to disease progression. The *CASP9*-associated risk model demonstrates strong prognostic utility and highlights apoptosis as a promising therapeutic axis in ccRCC.

**Supplementary Information:**

The online version contains supplementary material available at 10.1007/s12672-025-03798-0.

## Introduction

Globally, renal cell carcinoma (RCC) ranks as the 14th most frequently diagnosed cancer, with over 400,000 new cases reported in 2020 [1]. The incidental detection rate of RCC has risen significantly due to the widespread use of abdominal imaging. Clear cell renal cell carcinoma (ccRCC), accounting for approximately 80% of RCC cases, is primarily driven by mutations in the *VHL* gene [2]. Over the past few decades, advancements in understanding the biology of ccRCC have led to improved therapeutic strategies and enhanced patient survival. Beyond surgical intervention, adjuvant and targeted therapies have been increasingly employed in patients with metastatic RCC. The emergence of immune checkpoint inhibitors (ICIs) has substantially improved overall survival in advanced RCC [3]. Nevertheless, many patients still develop locally advanced or distant metastases [4], highlighting an unmet need for better prognostic outcomes. Consequently, identifying novel therapeutic targets remains a critical area of investigation.​.

Apoptosis, a genetically regulated form of active cell death, is also referred to as programmed cell death [5]. It serves as a critical mechanism in multicellular organisms to maintain homeostasis by eliminating damaged [6], senescent, or superfluous cells [7]. A hallmark of apoptosis is the release of cytochrome c from mitochondria, a process tightly controlled by the balance between pro-apoptotic and anti-apoptotic proteins within the *BCL2* family. This cascade involves initiator caspases (*CASP8*, *CASP9*, and *CASP10*) and executioner caspases (caspase-3, -6, and − 7). Ultimately, apoptosis leads to nuclear membrane rupture mediated by caspase-6, proteolytic cleavage of intracellular proteins, cellular blebbing [5], and fragmentation of genomic DNA into nucleosomal units [8]. The role of apoptosis in cancer presents a paradox: it acts as a tumor-suppressive mechanism by eliminating malignant or premalignant cells. This is exemplified by Kurtova et al., who showed that chemotherapy-induced apoptosis inhibits tumor regrowth in bladder cancer models [9]. Conversely, apoptosis can also promote tumorigenesis. Ford et al. demonstrated that apoptotic cells in B-cell lymphoma contribute to tumor progression by inducing macrophage polarization and establishing a pro-regenerative, immunosuppressive microenvironment [10]. ​Despite its significance, the precise role of apoptosis in ccRCC remains poorly defined. Systematic investigations are urgently needed to characterize apoptotic dynamics in ccRCC and elucidate its underlying mechanisms. Such studies could provide critical insights for optimizing therapeutic strategies and improving patient prognosis [11].​

In the current study, we systematically characterized the apoptotic landscape in ccRCC and established a significant correlation between the key apoptotic gene *CASP9* and patient prognosis. By constructing a robust prognostic model based on differentially expressed genes between *CASP9*-high and *CASP9*-low tumor cells, we demonstrated its strong predictive performance. Our findings not only elucidate the complex role of apoptosis in ccRCC pathogenesis but also provide a foundation for future mechanistic investigations into the molecular pathways linking *CASP9* dysregulation to renal cancer progression. This work highlights apoptosis as a potential therapeutic target and prognostic biomarker in ccRCC, with important implications for clinical management.

Previous studies have explored apoptosis in renal cancer using bulk RNA-seq or limited single-cell datasets, but most did not fully address the spatial heterogeneity or genetic causality of apoptosis-related pathways [12]. At present, few studies have combined single-cell, spatial transcriptomic, and genetic evidence to comprehensively evaluate the role of *CASP9* in ccRCC [13]. In this study, we sought to integrate multi-omics data to investigate the potential significance of *CASP9*-driven apoptotic programs in malignant cell states and their interactions with the tumor immune microenvironment, and further developed a prognostic model with potential clinical relevance. We hope that these explorations may complement existing literature to some extent and provide a reference for future mechanistic studies and translational applications.

## Method

### Integrated single-cell and spatial transcriptomic analysis based on apoptosis signatures

Single-cell RNA-seq data from renal cell carcinoma samples were obtained from three GEO datasets: GSE131685, GSE152938, and GSE171306. Raw expression matrices and metadata were processed using Seurat (v4.0). After merging the datasets, normalization was performed using the LogNormalize method, followed by identification of highly variable genes, principal component analysis (PCA), clustering, and visualization using t-SNE and UMAP. Cells were annotated based on known markers.

Cancer cells were isolated and scored for apoptosis activity using a curated gene set and the AddModuleScore() function. Based on the median score, cancer cells were classified into apoptosishighMali and apoptosislowMali groups. All cells were merged into a combined Seurat object and randomly downsampled (*n* = 3,000) for cell-cell communication analysis using CellChat. CellChatDB entries related to “Secreted Signaling” and “Cell-Cell Contact” were used to infer ligand-receptor interactions. Communication probabilities and significance values were computed and visualized using the ktplots package, focusing on interactions between Mali cells and macrophages, monocytes, and cycling cells.

To further explore transcriptional transitions within the Mali population, pseudotime trajectory analysis was performed following Harmony integration. UMAP embeddings and rank-normalized Harmony components were used for QP (Quantum Polarization) trajectory inference with a custom vector-based approach to identify directional progression trends based on apoptosis signature scores.

For spatial deconvolution, apoptosis-grouped single-cell data were used as a reference for RCTD analysis of 10x Visium spatial transcriptomic data. A custom Rcpp-based function converted sparse count matrices to dense matrices, and cell types including apoptosishighMali, apoptosislowMali, macrophages, monocytes, and cycling cells were used in the reference construction. SpatialFeaturePlot was applied to visualize the spatial distributions of these populations across tissue sections, enabling investigation of their localization patterns and microenvironmental associations.

### Spatial interaction network and multiscale modeling of apoptosis-high cancer cells

To further investigate the spatial organization and interactions of apoptosis-high cancer cells, we constructed homotypic and heterotypic cell-cell interaction networks based on spatial transcriptomic data deconvoluted via RCTD. The deconvolution matrix was derived from prior single-cell grouping using apoptosis signature scores, including cell types.

For homotypic networks, we selected spots where the proportion of ApoptosishighMali and ApoptosislowMali cells exceeded 0.1. Spot-to-spot proximity was determined using a K-nearest neighbor (KNN) algorithm (k = 6, max distance = 200 μm). For each spot, the number of neighboring spots also enriched in ApoptosishighMali was computed and defined as the degree of spatial clustering. These were visualized as spatial networks overlaid on tissue histology using scaled low-resolution 10x Visium images.

For heterotypic networks, we focused on the spatial relationship between ApoptosishighMali and macrophages. Spots were filtered by expression thresholds, and spatial proximity-based edges were drawn between different cell types. Line segments represented potential cell-cell interactions, and their spatial coordinates were registered to the tissue image. Additionally, a neighbor enrichment score was computed to quantify the local abundance of immune cells around apoptosis-high cancer cells.

To investigate spatial signal propagation through the *SPP1–CD44* axis, we applied the commot Python package for ligand–receptor (LR) flow modeling. The ligand–receptor pair of interest was defined as LR = np.array([[‘*SPP1*’, ‘*CD44*’, ‘*SPP1*’]], dtype = str). Using spatial transcriptomic data and RCTD-derived cell-type proportions as inputs, commot inferred the spatial dynamics and directionality of intercellular communication mediated by *SPP1* signaling. The analysis produced directed communication vectors across the tissue, visualized as arrows indicating the flow of ligand activity from sender to receiver locations. This enabled quantification and mapping of signal propagation, particularly between ApoptosishighMali cells and target populations such as *CD44*-expressing macrophages and monocytes, providing insight into spatially resolved tumor–immune interactions.

### Single-cell RNA sequencing analysis for CASP9

Cancer cells were extracted and stratified into *CASP9*-high and *CASP9*-low groups based on *CASP9* expression levels. Cell-cell communication analysis was performed using the CellChat package, focusing on secreted signaling and cell–cell contact pathways. Key ligand-receptor interactions were visualized using the ktplots package. To investigate developmental trajectories, Harmony was used for batch effect correction, and pseudotime inference was performed using the Vector.R algorithm to compute polarization vectors and directional transitions.

### Spatial transcriptomic analysis and spatial cell interaction mapping of CASP9⁺ cells

Spatial transcriptomic data from human ccRCC tissues were processed using the Seurat package (v4). Raw data were loaded using the Load10X_Spatial() function from the filtered_feature_bc_matrix.h5 file. Only “on-tissue” spots were retained based on tissue annotation. To reduce technical noise, mitochondrial and ribosomal genes (e.g., those matching “^*MT-*”, “^*RPS*”, “^*RPL*”, or “^*MRP*”) were excluded. Genes expressed in fewer than 10 spatial barcodes, as well as spots with fewer than 300 detected genes or fewer than 500 total counts, were also removed.

After quality control, data normalization was performed using both the SCTransform and LogNormalize methods. Dimensionality reduction was performed with PCA and UMAP using the top 30 principal components, followed by clustering using the shared nearest neighbor (SNN) method. Spatial expression of *CASP9* was visualized using SpatialFeaturePlot() to explore its distribution within the tumor microenvironment.

To estimate cell type composition across spatial spots, we applied RCTD (Robust Cell Type Decomposition), using manually curated scRNA-seq data as reference. A custom Rcpp-based as_matrix() function was used to convert the sparse count matrix into a dense format, and spatial deconvolution was performed using create.RCTD() and run.RCTD() in full doublet mode. The resulting cell type proportions were integrated into the spatial metadata.

Based on RCTD results, spatial interaction networks were constructed to characterize homotypic and heterotypic relationships involving *CASP9*^+^ cells. For homotypic interactions, we selected spots with *CASP9*^+^ cell proportions > 0.1 and built spatial neighbor networks using KNN (k = 6, distance ≤ 200). The number of *CASP9*^+^ neighbors per spot was defined as the spatial degree and visualized on the tissue image. For heterotypic networks, we focused on interactions between *CASP9*^+^ cells and *CD8*^+^*T* cells, identifying spatial neighbors with proportions > 0.1. Interactions were visualized as line segments linking the two cell types, and a normalized neighbor enrichment score was computed to quantify macrophage density around *CASP9*^+^ spots.

### Summary-based Mendelian randomization (SMR) analysis

We performed a SMR analysis to identify putative causal genes associated with renal cell carcinoma. Genome-wide association summary statistics for kidney cancer (ID: ukb-b-1316) were obtained from the OpenGWAS database. European population data were used as the reference panel to estimate linkage disequilibrium (LD) structure. After formatting the GWAS summary data into the required .ma file (mygwas.ma), we conducted SMR analysis using the SMR software. Genes identified by SMR were subsequently intersected with a predefined APOPTOSIS gene set to prioritize functionally relevant candidates. *CASP9* emerged as a key gene through this intersection. To visualize the association, we generated regional association plots (locus plots) and SMR scatter plots depicting the relationship between SNP effect sizes from eQTL and GWAS datasets.

### Prognostic model construction based on CASP9-defined malignant cell states

To construct a prognostic model based on apoptosis-related malignant signatures, we first identified differentially expressed genes (DEGs) between apoptosis-high and apoptosis-low malignant cells using single-cell RNA sequencing data. Differential expression analysis was performed with the Seurat package (v4.0) using the FindMarkers function with a log fold change threshold of 0.5, and ribosomal genes (e.g., *RPS*/*RPL* family) were excluded to reduce noise. A total of five apoptosis-related genes were identified and used for model construction.

Bulk RNA-seq expression data and clinical survival information for 531 ccRCC patients from TCGA-KIRC were used as the training set. An independent validation set consisting of 101 samples from the E-MTAB-1980 cohort was also included. Log2 transformation was applied to the expression matrices, and batch effects between datasets were corrected using the ComBat algorithm from the sva package. After harmonization, univariate Cox regression was performed on the training set to screen survival-associated genes. Lasso-Cox regression was then applied to select the optimal gene subset, resulting in a 5-gene prognostic signature.

Risk scores were computed using gene expression and Lasso-derived coefficients. The model was trained using the DeepSurv deep learning framework with 1,000 epochs, and training was supervised using the TensorboardLogger. Predicted risk scores were applied to the validation set. Kaplan-Meier survival curves and time-dependent ROC analyses were used to evaluate model performance in both cohorts.

### Risk score evaluation and immune profiling

Clinical data including age, gender, TNM classification, and tumor stage were extracted from TCGA-KIRC and matched with risk scores. Univariate and multivariate Cox regression analyses were performed to identify independent prognostic factors, and forest plots were used to visualize hazard ratios and confidence intervals. Subgroup survival analysis was conducted in patients stratified by clinical characteristics (e.g., stage 2–3, female, M1, N1, age ≥ 65) to assess the predictive value of the risk score using Kaplan–Meier curves and log-rank tests. To explore the immune landscape, immune and stromal scores were estimated using MCPcounter and ESTIMATE via the IOBR package, while ssGSEA was applied to evaluate immune pathway activity. Boxplots and ridge plots were used to compare differences between high- and low-risk groups. Additionally, immune checkpoint gene expression was extracted and analyzed between the two groups.

## Result

### Heterogeneity of apoptosis-related malignant cell states

**Fig. 1:**
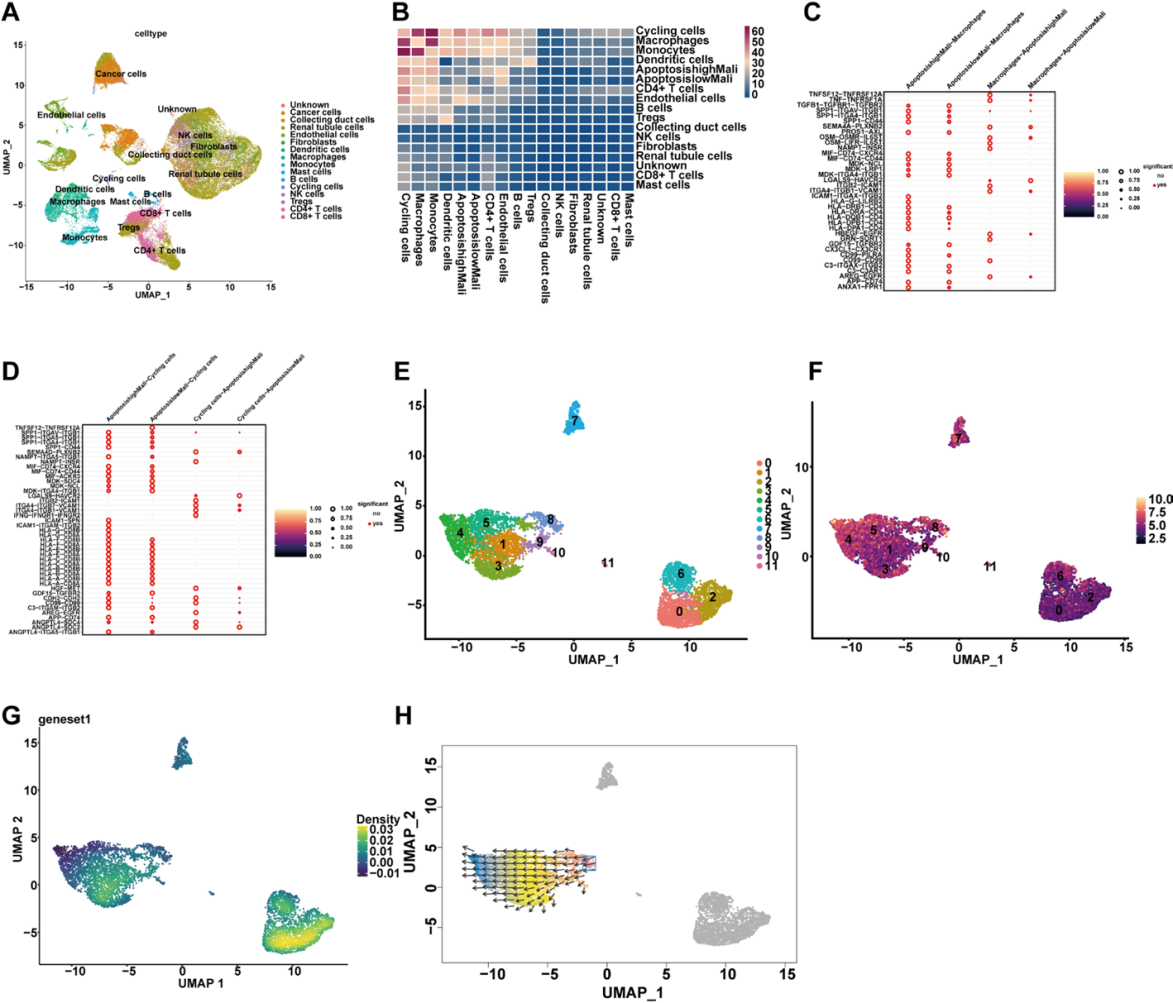
Single-cell analysis of apoptosis-related signatures in the tumor microenvironment. **A** UMAP plot displaying the distribution of annotated cell types. **B** Heatmap showing the overall cell–cell communication intensity between malignant cells with high or low apoptosis signature scores and various cell types. **C**,** D** Dot plots illustrating significant ligand–receptor interactions between high/low apoptosis malignant cells and macrophages **C** or cycling cells **D**. **E** UMAP showing re-clustering of malignant cells with high apoptosis signature scores. **F** UMAP visualization of apoptosis gene set scores in single cells, colored by enrichment level. **G** Density map of apoptosis gene set scores across the UMAP. **H** Pseudotime trajectory inferred by RNA velocity analysis, indicating potential developmental dynamics of high-apoptosis malignant cells

To investigate the apoptotic landscape of malignant cells in ccRCC, we first performed unsupervised clustering and annotation of integrated single-cell RNA sequencing data, identifying major cell populations including cancer cells, endothelial cells, fibroblasts, monocytes, macrophages, cycling cells, *CD4*^+^ and *CD8*^+^*T* cells, NK cells, dendritic cells, B cells, plasma cells, mast cells, renal tubular epithelial cells, and collecting duct cells (Fig. 1A). Using CellChat, we analyzed intercellular communication networks and found that apoptosishighMali and apoptosislowMali cancer cells exhibited markedly distinct interactions with components of the tumor microenvironment. Specifically, differences in communication intensities were observed between these two groups and cycling cells, macrophages, monocytes, *CD4*^+^*T* cells, and endothelial cells (Fig. 1B). Detailed analysis showed that malignant cells interacted with cycling cells through multiple pathways. The most significant included *TNFSF12–TNFRSF12A*,* TNF–TNFRSF1A*,* TGFB1–TGFBR1–TGFBR2*, and several SPP1-mediated axes (Fig. 1C). Similarly, macrophage interactions were mediated by several signaling axes. These included *TNFSF12–TNFRSF12A*, multiple SPP1-related pathways, as well as *SEMA4D–PLXNB2 and NAMPT–ITGA5–ITGB1* (Fig. 1D). To further explore heterogeneity within the apoptosis-high population, reclustering of apoptosishighMali cancer cells identified 11 transcriptionally distinct subclusters (Fig. 1E). Expression analysis of the apoptosis-related gene *CASP9* revealed higher expression levels in clusters 0, 2, and 6, and moderate expression in clusters 3 and 4, suggesting differential activation of apoptotic programs within these malignant subpopulations (Fig. 1F–G). Finally, pseudotime trajectory inference based on quantum polarization (QP) vectors indicated a continuous differentiation path within the mixed clusters. *CASP9* expression progressively increased along this trajectory, suggesting that apoptotic activity may be gradually acquired during malignant cell evolution (Fig. 1H).

### Spatial mapping and interaction networks reveal the microenvironmental localization and signaling of apoptosis-high malignant cells

**Fig. 2:**
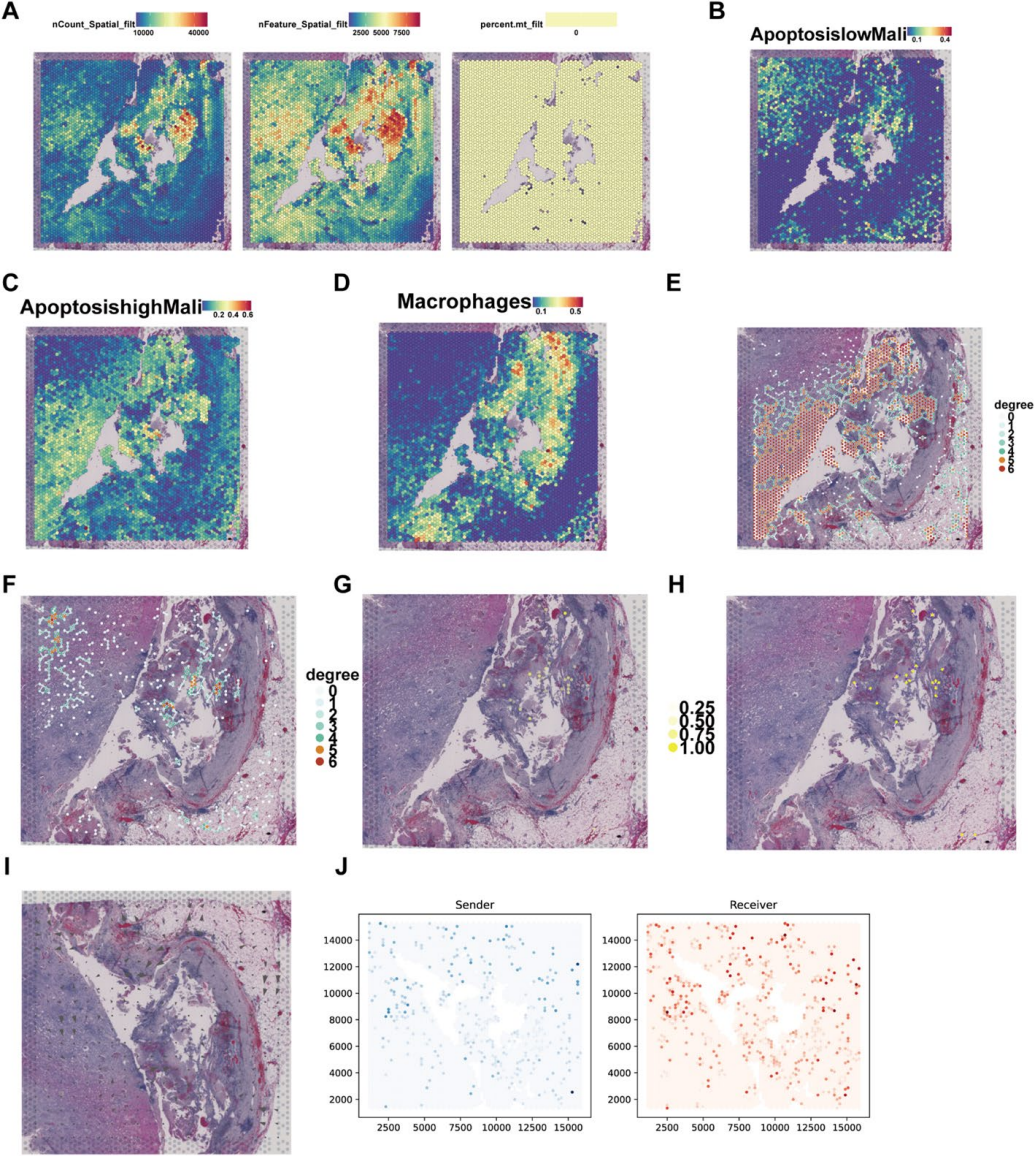
Spatial localization and intercellular communication analysis of apoptosis-high malignant cells. **A** Spatial quality control metrics of the spatial transcriptomics dataset. **B** Spatial distribution of apoptosis-low malignant cells. **C** Spatial distribution of apoptosis-high malignant cells. **D** Spatial distribution of macrophages. **E** Homotypic cell network of apoptosis-high malignant cells. **F** Homotypic cell network of apoptosis-low malignant cells. **G**, **H** Heterotypic cell network showing interactions between apoptosis-high malignant cells and macrophages. **I** Cell–cell communication (commot) flow inferred from spatial context. **J** Positive expression scores of communication roles (Sender and Receiver) based on commot analysis

To investigate the spatial distribution of apoptosis-defined malignant subpopulations, we performed spatial transcriptomic analysis using RCTD-based deconvolution. Figure 2A displays key quality control metrics, including total UMI counts (nCount_Spatial), number of detected genes (nFeature_Spatial), and the percentage of mitochondrial genes (percent.mt_filter) across tissue spots, ensuring data reliability. Apoptosis-high and apoptosis-low malignant cells exhibited distinct spatial localization patterns across the tumor sections (Fig. 2B, C). Notably, apoptosis-high cancer cells were enriched near the invasive front and adjacent stromal regions, whereas apoptosis-low cells were more diffusely distributed within the tumor core. In parallel, macrophages were found to preferentially localize in proximity to apoptosis-high malignant cells, suggesting potential spatial interaction (Fig. 2D). To assess homotypic spatial clustering, we constructed spatial proximity networks for apoptosis-high and apoptosis-low malignant cells separately. Apoptosis-high cells displayed dense local clustering with strong intra-group connectivity (Fig. 2E), while apoptosis-low cells exhibited more scattered distribution and weaker spatial coherence (Fig. 2F). This contrast in spatial organization underscores distinct microenvironmental associations between malignant cell subtypes.

We next examined heterotypic cell–cell interactions between apoptosis-high cancer cells and macrophages. The spatial interaction network revealed a high degree of co-localization and spatial connectivity between these two cell types (Fig. 2G, H), supporting potential intercellular communication in the tissue context. Consistent with this, ligand–receptor flow modeling via the commot framework indicated a directed signaling axis mediated by *SPP1–CD44*, with signal vectors originating from apoptosis-high malignant cells and targeting macrophage-enriched regions (Fig. 2I). Finally, sender–receiver mapping demonstrated that *SPP1* expression (Sender) was predominantly localized in tumor epithelial regions, while *CD44* (Receiver) was enriched in immune cell–dense zones, particularly around macrophages and monocytes (Fig. 2J). Senders are primarily located in the epithelial area, while Receivers are enriched in the surrounding stroma, revealing a spatially ordered intercellular communication pathway mediated by the *SPP1–CD44* axis. This spatially resolved sender–receiver pairing provides mechanistic support for functional ligand transmission from apoptosis-high cancer cells to *CD44*-expressing immune populations, reinforcing the critical role of the *SPP1–CD44* axis in mediating tumor–immune interactions.

### Single-cell and SMR analysis demonstrated the critical role of casp9 in the immune environment and pathogenesis

**Fig. 3:**
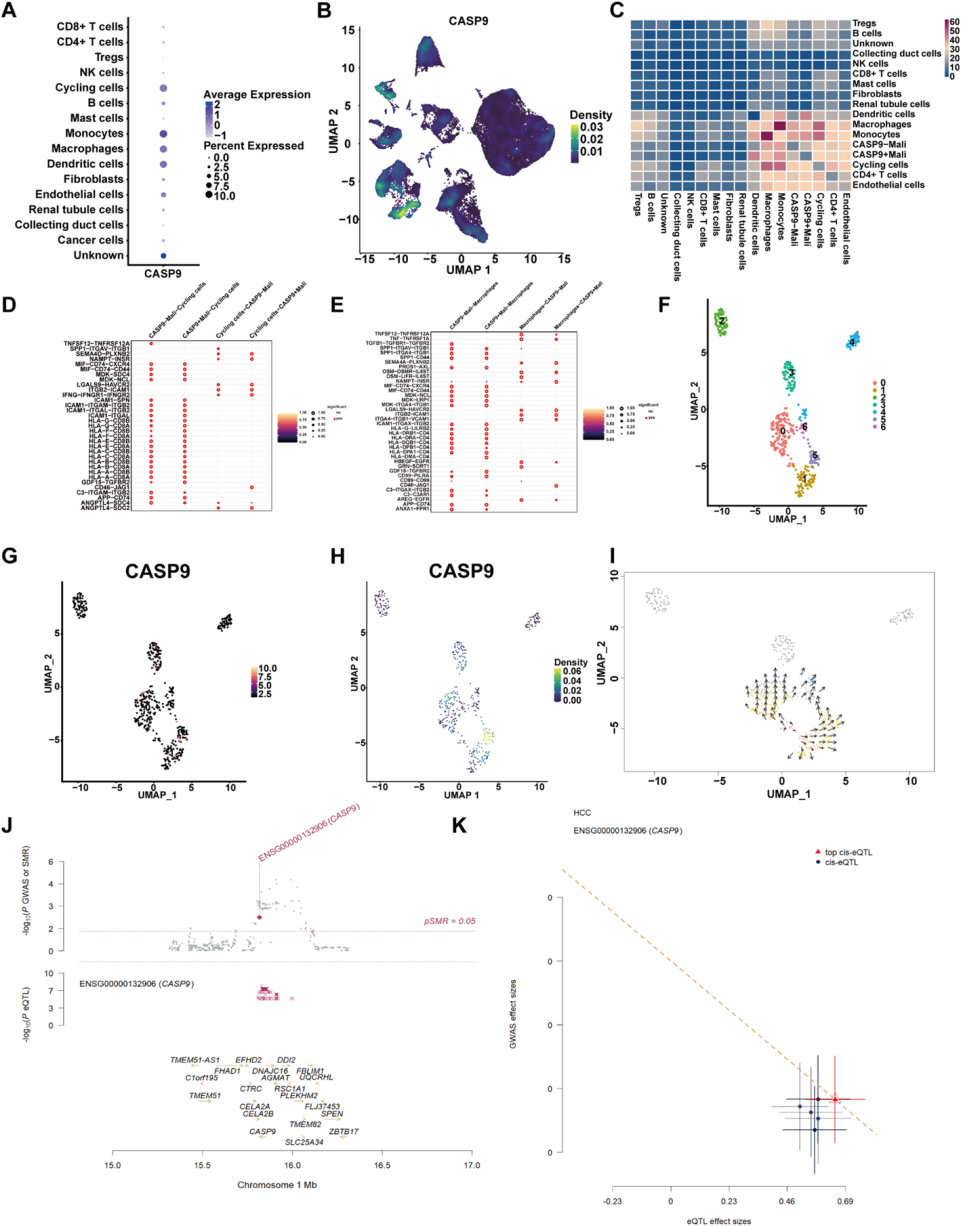
Single-cell transcriptomic analysis of CASP9 expression and cell-cell communication in the tumor microenvironment. **A** Dot plot showing CASP9 average expression and percent of expressing cells across cell types. **B** UMAP plot depicting the spatial expression of CASP9. **C** Heatmap showing the overall communication intensity between CASP9-high/low malignant cells and other cell types. **D**, **E** Dot plots showing ligand–receptor interactions between CASP9-high/low malignant cells and cycling cells **D** or macrophages **E**. **F** Re-clustering of CASP9-high malignant cells. **G** CASP9 expression level in each subcluster. **H** Density of CASP9-expressing cells in the UMAP space. **I** Pseudotime trajectory analysis of CASP9-high malignant cells. **J** Summary-data-based Mendelian Randomization (SMR) analysis integrating GWAS and cis-eQTL data. **K** HEIDI (heterogeneity in dependent instruments) plot showing the relationship between eQTL effect sizes and GWAS effect sizes for CASP9

To explore the cellular localization and functional implications of *CASP9* expression, *CASP9* expression was found to be significantly elevated in malignant cells compared to non-malignant populations (Fig. 3A). Notably, *CASP9* was also highly expressed in macrophages, monocytes, and cycling cells, suggesting a broader role in the tumor microenvironment. UMAP visualization confirmed that *CASP9* expression was spatially enriched in malignant regions (Fig. 3B). To evaluate the functional impact of *CASP9* expression on cell-cell communication, we performed CellChat analysis comparing *CASP9*-high and *CASP9*-low malignant cells. The results revealed markedly distinct interaction patterns with macrophages, consistent with prior gene set-based analyses (Fig. 3C). Further analysis revealed multiple signaling pathways connecting CASP9-high malignant cells and cycling cells. The key ligand–receptor pairs included *TNFSF12–TNFRSF12A* and *SPP1–ITGAV–ITGB1* (Fig. 3D). Interactions with macrophages were dominated by *TNFSF12–TNFRSF12A*,* TNF–TNFRSF1A*,* TGFB1–TGFBR1–TGFBR2*, and several SPP1-related pathways (Fig. 3E), corroborating previous findings based on apoptosis-related signaling networks. To gain further insight into malignant heterogeneity, *CASP9*-high malignant cells were reclustered into six distinct subgroups (Fig. 3F). *CASP9* expression was predominantly localized in clusters 0, 4, and 5 (Fig. 3G–H), suggesting transcriptional sub-specialization within the *CASP9*-high population. Pseudotime trajectory analysis revealed a progressive increase in *CASP9* expression along the developmental axis of malignant cell differentiation (Fig. 3I), consistent with its enrichment in late-stage or more aggressive tumor subsets.

To explore the genetic regulatory mechanisms linking *CASP9* expression to renal cancer risk, we conducted SMR analysis integrating GWAS and eQTL datasets. As shown in Fig. 3J, *CASP9* (ENSG00000132906) was identified as a significant candidate gene with a notable pSMR value below the 0.05 threshold, indicating a potential causal relationship between its genetically regulated expression and renal cancer susceptibility. Nearby genes such as *TMEM51-AS1* and *DDI2* were not significantly associated, highlighting the specificity of *CASP9*. Figure 3K further validates this association through a HEIDI (heterogeneity in dependent instruments) test. The lead cis-eQTL for *CASP9* (marked in red) showed a strong and consistent effect size direction when compared with GWAS signals. The alignment of eQTL and GWAS effect sizes suggests that the same genetic variant likely mediates both *CASP9* expression and disease risk, thereby excluding horizontal pleiotropy. Collectively, these results support the hypothesis that *CASP9* expression is genetically regulated and may act as a functional driver of renal cancer development, providing genetic evidence for its role in tumorigenesis.

### CASP9-high malignant cells exhibit spatial proximity to macrophages and form distinct multicellular interaction modules

**Fig. 4:**
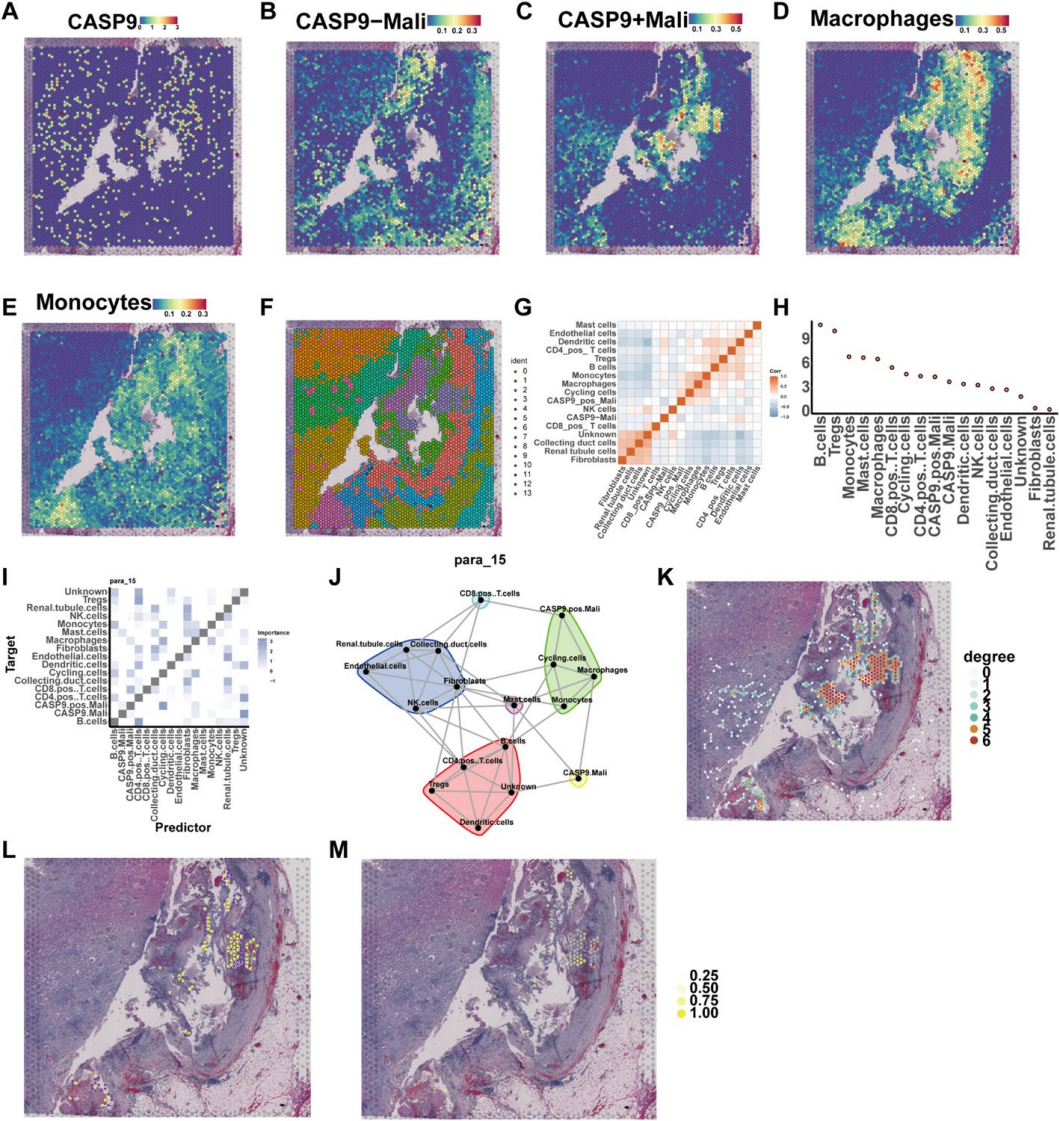
Spatial analysis of CASP9 expression and intercellular communication in spatial transcriptomics. **A** Spatial expression of CASP9. **B–E** Spatial distribution of CASP9-high malignant cells **B**, CASP9-low malignant cells **C**, macrophages **D**, and monocytes **E**. **F** Clustering results of spatial domains. **G**, **H** mistyR spatial interaction analysis: **G** Heatmap showing intercellular association scores across spatial domains. **H** Dot plot showing the correlation between CASP9-high malignant cells and other cell types. **I**,** J** para (partitioning-based spatial regression analysis): **I** Heatmap of predictor-target importance values. **J** Network diagram showing key regulatory influences between cell types. **K** Homotypic cell network of CASP9-high malignant cell. **L**, **M** Heterotypic cell networks between CASP9-high malignant cells and macrophages: **L** Shows network edges between the two cell types. **M** Highlights enriched macrophage nodes (mycell2) interacting with malignant cells

To further investigate the spatial organization and intercellular associations of *CASP9*-high malignant cells, the spatial localization of *CASP9* expression revealed distinct clustering patterns (Fig. 4A), with *CASP9*-high and *CASP9*-low malignant cells occupying separate territories. Spatial mapping of malignant cells with high and low *CASP9* expression, along with macrophages and monocytes, showed a clear separation between high and low expression groups (Fig. 4B–E). *CASP9*-high malignant cells were predominantly co-localized with macrophages, while monocytes displayed less spatial specificity. These findings suggest a stronger spatial coupling between *CASP9*-high tumor cells and macrophages within the tumor microenvironment. Subsequent clustering analysis indicated that *CASP9*-high, *CASP9*-low malignant cells, and macrophages were primarily enriched in clusters 0, 6, and 11, respectively (Fig. 4F), further supporting the spatial segregation.

To quantify cell-cell interaction dynamics, we applied MistyR modeling. The results showed significant differences in the contextual associations of *CASP9*-high and *CASP9*-low malignant cells with cycling cells and macrophages (Fig. 4G), highlighting the influence of *CASP9* expression on microenvironmental interactions. In particular, Fig. 4H revealed the relative contribution of each cell type to the spatial context of *CASP9*-high cells. Macrophages emerged as the one of the dominant contributors, reinforcing their spatial and functional relevance. Using paraview-based reconstruction, we observed that *CASP9*-high malignant cells formed a tightly connected spatial module with macrophages, monocytes, and cycling cells (Fig. 4J–J), indicating the existence of a coordinated multicellular niche. Homotypic interaction networks further demonstrated that *CASP9*-positive malignant cells tend to aggregate within defined regions (Fig. 4K), whereas heterotypic interaction mapping (Fig. 4L–M) highlighted the strong interaction intensity and spatial alignment between *CASP9*-high malignant cells and macrophages, validating their cooperative role in the spatial tumor structure.

### Deep learning-based survival modeling identifies CASP9-associated prognostic signature in CcRCC

**Fig. 5:**
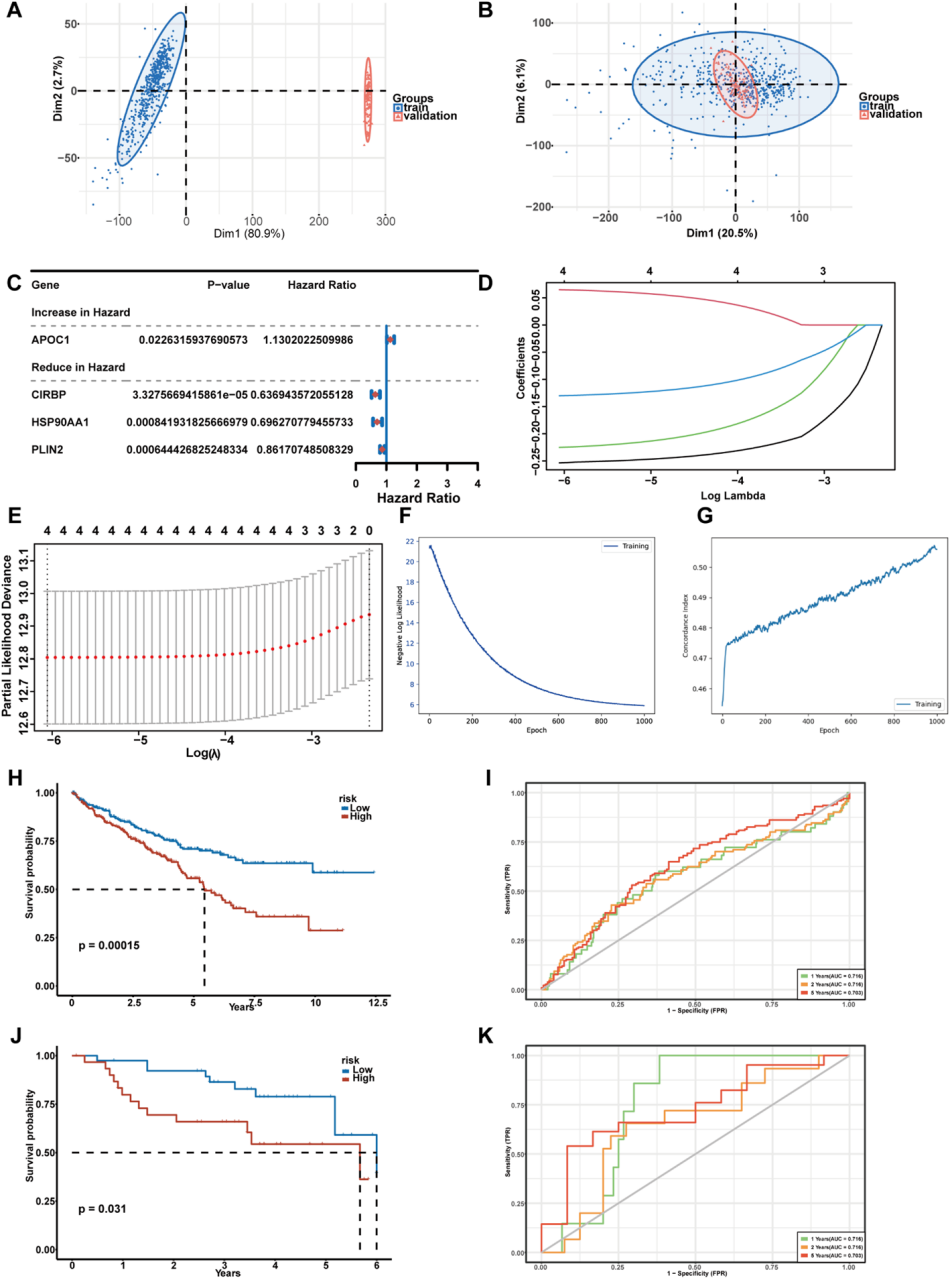
Development and validation of a CASP9-associated prognostic signature in ccRCC. **A** Dimensionality reduction plot of the training set before batch correction. **B** Distribution of training and validation sets after batch correction. **C** Forest plot of univariate Cox regression results. Genes with significant associations to survival. **D** LASSO regression coefficient paths across a sequence of log(λ) values. **E** Selection of the optimal λ value based on minimum cross-validation error during LASSO regression. **F** Training loss curve of the DeepSurv model. **G** Concordance index (C-index) curve over epochs during DeepSurv model training. **H** Kaplan-Meier survival curve of high- vs. low-risk groups in the training set. **I** ROC curves at 1, 3, and 5 years in the training set. **J** Kaplan-Meier survival curve of high- vs. low-risk groups in the validation set. **K** ROC curves at 1, 3, and 5 years in the validation set

To construct a *CASP9*-associated prognostic model, we first performed dimensionality reduction on the training dataset, which revealed distinct separation of samples prior to batch correction (Fig. 5A). After batch correction, the training and validation sets were well integrated, indicating effective removal of batch effects (Fig. 5B). Univariate Cox regression analysis identified several survival-related genes for downstream modeling (Fig. 5C). LASSO regression was used to narrow down prognostic features, with the coefficient paths visualized over a range of log(λ) values (Fig. 5D), and the optimal λ selected based on minimum cross-validation error (Fig. 5E). We then trained a DeepSurv model using the selected gene set. The training loss progressively decreased (Fig. 5F), and the concordance index (C-index) steadily increased across epochs (Fig. 5G), indicating robust model convergence. In the training cohort, Kaplan–Meier analysis showed that patients in the high-risk group had significantly worse survival outcomes (*p* = 0.00015) (Fig. 5H), and time-dependent ROC curves demonstrated favorable predictive accuracy (Fig. 5I). The model’s performance was further validated in an independent cohort, showing significant survival differences (*p* = 0.031) (Fig. 5J) and consistent predictive power across timepoints (Fig. 5K).

### CASP9-based risk score serves as an independent prognostic indicator across diverse clinical subgroups

**Fig. 6:**
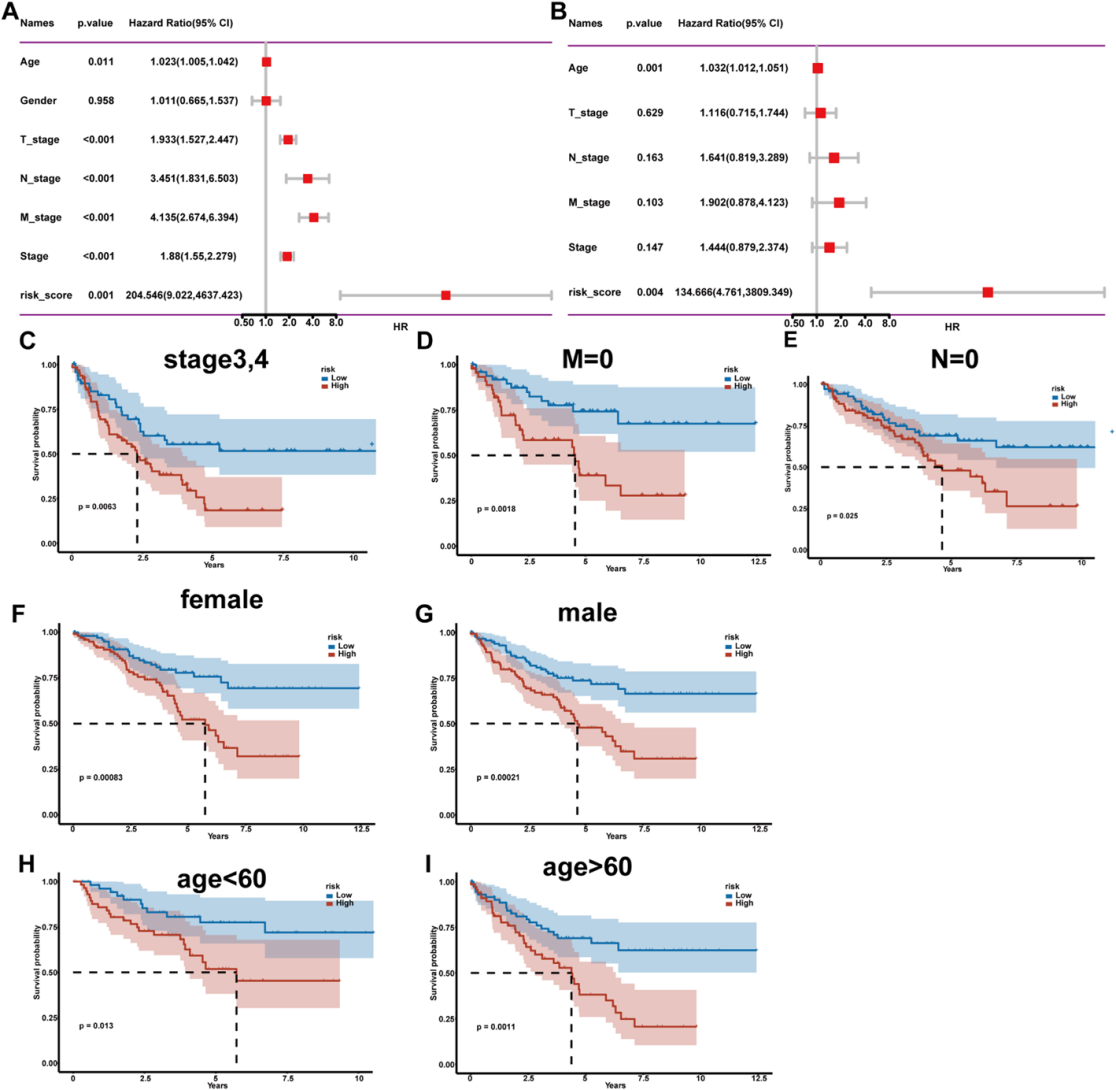
Univariate and multivariate Cox analysis of the prognostic model and subgroup survival validation. **A** Univariate Cox regression analysis of clinical variables and risk score. **B** Multivariate Cox regression analysis including clinical features and risk score. **C** Kaplan-Meier survival curve of patients with stage III–IV disease, stratified by risk score. **D** Kaplan-Meier survival analysis in patients with M0 stage (no distant metastasis). **E** Kaplan-Meier survival curve in patients with N0 stage (no lymph node metastasis). **F** Kaplan-Meier survival curve in female patients. **G** Kaplan-Meier survival curve in male patients. **H** Kaplan-Meier survival analysis in patients younger than 60 years. **I** Kaplan-Meier survival analysis in patients older than 60 years. HR were calculated using Cox proportional hazards regression. Survival differences were evaluated by log-rank test

To evaluate the independent prognostic value of the *CASP9*-based risk score, univariate and multivariate Cox regression analyses were performed. In the univariate analysis, both TNM stage and the risk score were significantly associated with overall survival, with the risk score showing the highest hazard ratio (HR = 204.546, 95% CI: 9.022–4657.423, *p* < 0.001) (Fig. 6A). In the multivariate analysis, after adjusting for clinical features such as age, stage, and TNM classification, the risk score remained an independent prognostic factor (HR = 134.666, 95% CI: 7.761–3006.349, *p* = 0.004) (Fig. 6B). We further validated the robustness of the risk model across multiple clinical subgroups. High-risk patients consistently exhibited poorer prognosis in advanced-stage (stage III–IV), M0, and N0 subgroups (Fig. 6C–E). The model also showed predictive power across both genders (Fig. 6F, G) and different age groups (< 60 and ≥ 60 years; Fig. 6H, I), reinforcing its prognostic stability in diverse clinical backgrounds.

### Immune landscape associated with the risk model

**Fig. 7:**
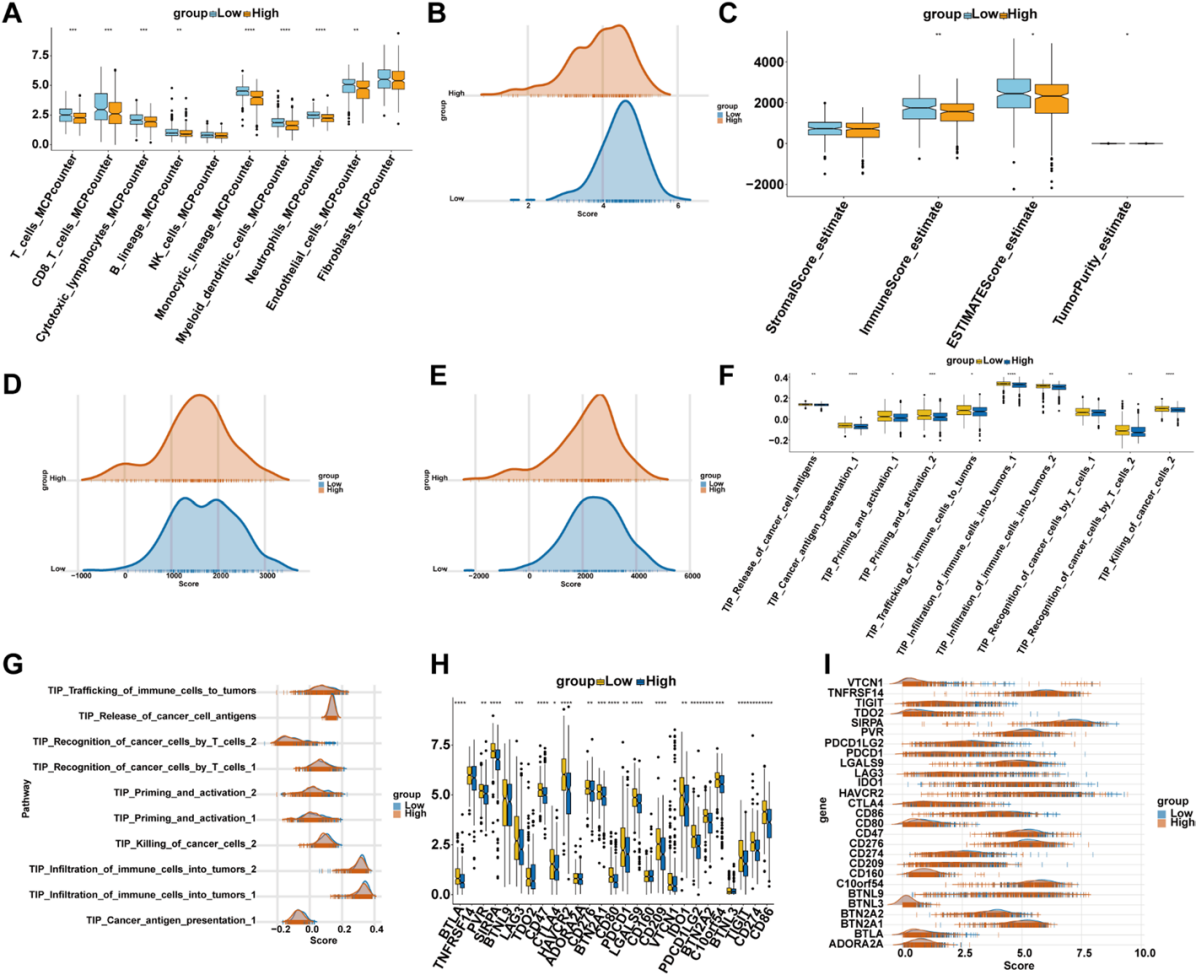
Immune landscape differences between high- and low-risk groups in KIRC. **A** MCP-counter estimated immune cell infiltration scores in high- and low-risk groups. **B** Ridge plot showing distribution of myeloid cells scores between groups. **C** ESTIMATE scores compared between groups. **D**,** E** Ridge plots showing the distribution of StromalScore **D** and ESTIMATEScore **E** between high- and low-risk groups. **F** Enrichment scores of TIP (Tracking Tumor Immunophenotype) pathways across risk groups, such as immune cell infiltration, activation, and antigen presentation. **G** Ridge plot visualization of representative TIP pathway scores in high- vs. low-risk groups. **H** Expression comparison of immune checkpoint genes (e.g., PDCD1, CTLA4, TIGIT, etc.) across groups. **I** Ridge plots displaying distribution of immune checkpoint gene expression in each group. All statistical analyses were performed using the Wilcoxon rank-sum test unless otherwise stated. Significance markers are indicated as follows: *p* < 0.05 (*), *p* < 0.01 (**), *p* < 0.001 (***), and *p* < 0.0001 (****). All analyses were performed on publicly available datasets (GEO and TCGA) as described in the Methods section; no additional biological replicates were performed

The low-risk group demonstrated a more active and immunologically enriched tumor microenvironment across multiple dimensions. The MCP-counter analysis revealed significantly higher infiltration scores of various immune cells in the low-risk group, including T cells, cytotoxic lymphocytes, B lineage cells, myeloid dendritic cells, neutrophils, and NK cells, indicating a more immunocompetent microenvironment (Fig. 7A). Ridge plots of myeloid lineage scores showed a pronounced left shift in the high-risk group, suggesting potential suppression or depletion of bone marrow-derived immune components in high-risk patients (Fig. 7B). ESTIMATE analysis indicated significantly higher ImmuneScore, StromalScore, and ESTIMATEScore in the low-risk group, reflecting a higher abundance of non-tumor components such as stromal and immune cells within the tumor microenvironment (Fig. 7C). Ridge plots of StromalScore and ESTIMATEScore consistently showed a rightward shift in the low-risk group, further supporting a more complex and immunologically active tissue composition (Fig. 7D, E). TIP (Tracking Tumor Immunophenotype) pathway enrichment analysis revealed stronger immune activation in the low-risk group, with pathways related to T cell infiltration, activation, and antigen presentation being significantly upregulated compared to the high-risk group (Fig. 7F). Ridge plots of representative TIP pathways confirmed their higher enrichment in the low-risk group (Fig. 7G). Analysis of immune checkpoint gene expression showed that most checkpoint molecules (e.g., *CD274*, *CD40*, *CD80*, *CD86*, *BTN2A2*) were more highly expressed in the low-risk group, while only a few (e.g., *PDCD1*, *TIGIT*) exhibited slight upregulation in the high-risk group. This suggests a higher immunotherapeutic potential in the low-risk subgroup (Fig. 7H). Ridge plots of immune checkpoint genes further validated these findings, showing a broader and higher expression distribution in the low-risk group, indicative of a more immunologically reactive state and potentially better response to immune checkpoint blockade therapy (Fig. 7I).

### Evaluation of CASP8 and CASP10 expression and spatial localization

To evaluate whether the observed effects were specific to *CASP9*, we examined the expression and spatial localization of other initiator caspases, including *CASP8* and *CASP10*. Dot plot analysis revealed that *CASP8* expression was enriched in CD4⁺ and CD8⁺ T cells but was low in malignant cells and macrophages (Figure S1-A). Spatial mapping further showed that *CASP8* expression was scattered across tissue sections without clear co-localization with macrophage-rich areas (Figure S1B, C). Similarly, *CASP10* was more highly expressed in monocytes, but its expression in malignant tumor cells was minimal, and its spatial distribution lacked overlap with macrophages (Figure S1D–F). These findings suggest that *CASP8* and *CASP10* do not share the spatial or cellular specificity observed for *CASP9*.

## Discussion

Apoptosis is a tightly regulated and evolutionarily conserved programmed cell death process that plays essential roles in normal physiology, including embryogenesis and adult tissue homeostasis [14]. It is also well-established as a critical tumor-suppressive mechanism. The resistance to apoptosis has been widely recognized as an acquired feature of cancer cells, conferring survival advantages that promote tumor progression and treatment resistance [15]. However, recent studies have revealed a paradoxical association between high apoptotic activity and poor prognosis in advanced cancers [16, 17].

Caspase-9 (*CASP9*) serves as a pivotal initiator caspase in the mitochondrial apoptosis pathway, whose activation relies on dimerization within the apoptosome complex (Apaf-1/cytochrome c platform) rather than conventional proteolytic cleavage [18–20]. Under physiological conditions, *CASP9* forms an active holoenzyme through CARD-CARD interactions between its N-terminal caspase recruitment domain (CARD) and Apaf-1 [21], subsequently activating downstream effector caspases (e.g., *caspase-3*) to execute apoptosis [18, 22]. The activity of *CASP9* is tightly regulated by multiple mechanisms, including phosphorylation-mediated inhibition, protein-protein interactions, and alternative splicing [23–25]. Accumulating evidence has linked reduced *CASP9* expression or functional mutations to chemotherapy resistance in cancers such as lung and bladder cancer [26–28].​​

Within this framework, our study adds a spatial and genetic perspective in ccRCC. Using single-cell and spatial transcriptomics, we observe that apoptosis-related programs are concentrated in malignant cells that preferentially co-localize with macrophage-rich regions, and ligand–receptor modeling implicates axises consistent with macrophage engagement. Complementing these observations, SMR analysis suggests a genetic contribution of *CASP9* to renal cancer risk. Together, these findings align with and extend prior reports by positioning *CASP9*-linked apoptotic states within a spatially organized, macrophage-associated niche in ccRCC, while motivating mechanistic testing of this axis in future studies.

Within this framework, our study adds a spatial and genetic perspective in ccRCC, which identified a specific interaction network between *CASP9*-high malignant cells and macrophages. This aligns with studies showing that apoptotic cells secrete chemotactic factors - including nucleotides [29, 30] and lipid molecules [31, 32] - to recruit macrophages to dying sites. Through receptor-ligand interactions, apoptotic cells induce M2-like polarization in macrophages, characterized by upregulated anti-inflammatory cytokines (*TGF-β1*,* IL-10*,* PGE2*) and downregulated pro-inflammatory cytokines (*TNFα*,* IL-1β*,* IL-12*) [33, 34]. The immunosuppressive effects of *IL-10* and *PGE2*, which suppress T-cell activity, further promote tumor progression [34]. Notably, macrophage metabolic reprogramming may represent a key mechanism underlying the protumorigenic effects of apoptotic cells.​

Mechanistically, our findings raise several hypotheses regarding how *CASP9*-high malignant cells may influence tumor progression. First, the enrichment of *CASP9*-expressing tumor cells near macrophage-dense regions, together with the activation of the *SPP1–CD44* axis, suggests that apoptosis-related programs may promote the recruitment and polarization of tumor-associated macrophages. Second, the metabolic reprogramming of macrophages in proximity to apoptotic tumor cells may further reinforce an immunosuppressive microenvironment, characterized by elevated IL-10 and PGE2 secretion [35].

Beyond its prognostic value in retrospective datasets, our *CASP9*-derived five-gene signature also holds potential for clinical translation. In practice, these genes could be measured through qPCR-based multi-gene panels or immunohistochemistry scoring systems, which are both widely accessible in pathology laboratories. Such approaches would allow stratification of patients into high- and low-risk groups in real time, facilitating more personalized management strategies. Moreover, integration of the signature with conventional clinical features (e.g., TNM staging, histological grading) may further enhance its predictive utility. Prospective validation in multi-center cohorts and the establishment of standardized assay protocols will be essential steps before implementation in clinical decision-making.

The value of our study lies primarily in its integrative methodology and potential translational implications. Previous investigations in ccRCC have provided valuable insights into tumor–immune crosstalk using either single-cell or spatial transcriptomic profiling [36], but these studies did not directly incorporate genetic evidence to support causal drivers. By combining scRNA-seq, spatial transcriptomics, and SMR, our work offers a multidimensional perspective. This integrative strategy enabled us to not only map *CASP9*-high malignant states within macrophage-rich niches, but also to demonstrate a genetic contribution of *CASP9* to renal cancer susceptibility.

Our comparative analysis of different initiator caspases reinforces the specificity of *CASP9* in apoptosis-associated malignant cell states. Unlike *CASP8* and *CASP10*, which were predominantly expressed in T cells and monocytes respectively, *CASP9* showed strong enrichment in malignant cells and clear spatial proximity to macrophages. This unique localization pattern supports *CASP9* as a central mediator of tumor–macrophage interactions in ccRCC. While other initiator caspases undoubtedly play important roles in apoptosis under different contexts, their limited expression in malignant cells and lack of spatial co-localization with macrophages suggest that they are unlikely to influence the spatial and prognostic conclusions of our study.

Our study also has certain limitations. While our bioinformatic analyses provide compelling insights, these findings require rigorous external validation. In particular, additional in vitro experiments and, where feasible, in vivo systems are necessary to confirm the robustness of our conclusions. Moreover, although the *CASP9*-derived five-gene signature demonstrates strong prognostic value across independent datasets, its clinical applicability remains to be established. Further research is required to validate this signature using standardized assays (e.g., qPCR panels or IHC scoring) and to assess its performance in prospective, multi-center cohorts. Notably, the molecular mechanisms underlying the protumorigenic effects of apoptosis remain incompletely understood and warrant further investigation to elucidate the complex interplay between apoptotic pathways and tumorigenesis.

In conclusion, our integrative single-cell and spatial transcriptomic analyses suggest that apoptosis-related gene sets are upregulated in tumor cells and closely interact with tumor-associated macrophages. In particular, the apoptotic gene *CASP9* appears to play an important role in defining malignant cell states and their spatial associations with the immune microenvironment. SMR analysis further indicates a potential genetic contribution of *CASP9* to ccRCC susceptibility. Moreover, the five-gene signature derived from *CASP9*-stratified tumor cells shows promising prognostic value across independent cohorts. Nevertheless, these findings should be interpreted with caution, as they require rigorous experimental validation in both in vitro and in vivo systems. Future work should also explore the feasibility of translating the *CASP9*-based prognostic model into clinically applicable tools, such as qPCR panels or IHC-based assays, to improve risk stratification and personalized treatment strategies in ccRCC.

## Supplementary Information

Below is the link to the electronic supplementary material.

Supplementary Material 1.

## Data Availability

All datasets used in this study are publicly available. Single-cell RNA sequencing data can be accessed from the Gene Expression Omnibus (GEO) under accession numbers GSE131685, GSE152938, and GSE171306. Bulk RNA-seq data and corresponding clinical information were obtained from The Cancer Genome Atlas (TCGA) database (https://portal.gdc.cancer.gov/). All custom code used for data processing, statistical analysis, and visualization has been uploaded to GitHub (https://github.com/zengwithhands122317/code2025.9), ensuring full reproducibility of the results.

## References

[1] Bukavina L, et al. Epidemiology of renal cell carcinoma: 2022 update. Eur Urol. 2022;82:529–42. 10.1016/j.eururo.2022.08.019. 36100483 10.1016/j.eururo.2022.08.019

[2] Moch H, et al. The 2022 world health organization classification of tumours of the urinary system and male genital Organs-Part A: Renal, Penile, and testicular tumours. Eur Urol. 2022;82:458–68. 10.1016/j.eururo.2022.06.016. 35853783 10.1016/j.eururo.2022.06.016

[3] Chen YW, et al. Treatment landscape of renal cell carcinoma. Curr Treat Options Oncol. 2023;24:1889–916. 10.1007/s11864-023-01161-5. 38153686 10.1007/s11864-023-01161-5 PMC10781877

[4] Vasudev NS, et al. Challenges of early renal cancer detection: symptom patterns and incidental diagnosis rate in a multicentre prospective UK cohort of patients presenting with suspected renal cancer. BMJ Open. 2020;10:e035938. 10.1136/bmjopen-2019-035938. 32398335 10.1136/bmjopen-2019-035938 PMC7223292

[5] Devis-Jauregui L, Eritja N, Davis ML, Matias-Guiu X. Llobet-Navàs, D. Autophagy in the physiological endometrium and cancer. Autophagy. 2021;17:1077–95. 10.1080/15548627.2020.1752548. 32401642 10.1080/15548627.2020.1752548 PMC8143243

[6] Xue Q, et al. Copper metabolism in cell death and autophagy. Autophagy. 2023;19:2175–95. 10.1080/15548627.2023.2200554. 37055935 10.1080/15548627.2023.2200554 PMC10351475

[7] Bertheloot D, Latz E, Franklin BS. Necroptosis, pyroptosis and apoptosis: an intricate game of cell death. Cell Mol Immunol. 2021;18:1106–21. 10.1038/s41423-020-00630-3. 33785842 10.1038/s41423-020-00630-3 PMC8008022

[8] Fraser A, Evan G. A license to kill. Cell. 1996;85:781–4. 10.1016/s0092-8674(00)81005-3. 8681372 10.1016/s0092-8674(00)81005-3

[9] Kurtova AV, et al. Blocking PGE2-induced tumour repopulation abrogates bladder cancer chemoresistance. Nature. 2015;517:209–13. 10.1038/nature14034. 25470039 10.1038/nature14034 PMC4465385

[10] Ford CA, et al. Oncogenic properties of apoptotic tumor cells in aggressive B cell lymphoma. Curr Biology: CB. 2015;25:577–88. 10.1016/j.cub.2014.12.059. 25702581 10.1016/j.cub.2014.12.059 PMC4353688

[11] Bai F, et al. Integrated analysis reveals crosstalk between pyroptosis and immune regulation in renal fibrosis. Front Immunol. 2024;15:1247382. 10.3389/fimmu.2024.1247382. 38343546 10.3389/fimmu.2024.1247382 PMC10853448

[12] Yu Z, et al. Integrative Single-Cell analysis reveals transcriptional and epigenetic regulatory features of clear cell renal cell carcinoma. Cancer Res. 2023;83:700–19. 10.1158/0008-5472.Can-22-2224. 36607615 10.1158/0008-5472.CAN-22-2224 PMC9978887

[13] Marques I, et al. Influence of survivin (BIRC5) and caspase-9 (CASP9) functional polymorphisms in renal cell carcinoma development: a study in a Southern European population. Mol Biol Rep. 2013;40:4819–26. 10.1007/s11033-013-2578-3. 23645041 10.1007/s11033-013-2578-3

[14] Moyer A, Tanaka K, Cheng EH. Apoptosis in cancer biology and therapy. Annu Rev Pathol. 2025;20:303–28. 10.1146/annurev-pathmechdis-051222-115023. 39854189 10.1146/annurev-pathmechdis-051222-115023

[15] Hanahan D, Weinberg RA. Hallmarks of cancer: the next generation. Cell. 2011;144:646–74. 10.1016/j.cell.2011.02.013. 21376230 10.1016/j.cell.2011.02.013

[16] Villar E, et al. bcl-2 expression and apoptosis in primary and metastatic breast carcinomas. Tumour Biology: J Int Soc Oncodevelopmental Biology Med. 2001;22:137–45. 10.1159/000050608. 10.1159/000050608 11275791

[17] Feng X, et al. Dying glioma cells Establish a proangiogenic microenvironment through a caspase 3 dependent mechanism. Cancer Lett. 2017;385:12–20. 10.1016/j.canlet.2016.10.042. 27826040 10.1016/j.canlet.2016.10.042 PMC5323266

[18] Bratton SB, Salvesen GS. Regulation of the Apaf-1-caspase-9 apoptosome. J Cell Sci. 2010;123:3209–14. 10.1242/jcs.073643. 20844150 10.1242/jcs.073643 PMC2939798

[19] Renatus M, Stennicke HR, Scott FL, Liddington RC, Salvesen GS. Dimer formation drives the activation of the cell death protease caspase 9. Proc Natl Acad Sci USA. 2001;98:14250–5. 10.1073/pnas.231465798. 11734640 10.1073/pnas.231465798 PMC64668

[20] Würstle ML, Laussmann MA, Rehm M. The central role of initiator caspase-9 in apoptosis signal transduction and the regulation of its activation and activity on the apoptosome. Exp Cell Res. 2012;318:1213–20. 10.1016/j.yexcr.2012.02.013. 22406265 10.1016/j.yexcr.2012.02.013

[21] Qin H, et al. Structural basis of procaspase-9 recruitment by the apoptotic protease-activating factor 1. Nature. 1999;399:549–57. 10.1038/21124. 10376594 10.1038/21124

[22] Stennicke HR, et al. Caspase-9 can be activated without proteolytic processing. J Biol Chem. 1999;274:8359–62. 10.1074/jbc.274.13.8359. 10085063 10.1074/jbc.274.13.8359

[23] Seifert A, Clarke PR. p38alpha- and DYRK1A-dependent phosphorylation of caspase-9 at an inhibitory site in response to hyperosmotic stress. Cell Signal. 2009;21:1626–33. 10.1016/j.cellsig.2009.06.009. 19586613 10.1016/j.cellsig.2009.06.009

[24] Shiozaki EN, et al. Mechanism of XIAP-mediated Inhibition of caspase-9. Mol Cell. 2003;11:519–27. 10.1016/s1097-2765(03)00054-6. 12620238 10.1016/s1097-2765(03)00054-6

[25] Vu NT, et al. HnRNP U enhances caspase-9 splicing and is modulated by AKT-dependent phosphorylation of HnRNP L. J Biol Chem. 2013;288:8575–84. 10.1074/jbc.M112.443333. 23396972 10.1074/jbc.M112.443333 PMC3605676

[26] Chee JL, et al. Wild-type and mutant p53 mediate cisplatin resistance through interaction and Inhibition of active caspase-9. Cell Cycle (Georgetown Tex. 2013;12:278–88. 10.4161/cc.23054. 23255126 10.4161/cc.23054 PMC3575457

[27] Liamarkopoulos E, et al. Caspase 8 and caspase 9 gene polymorphisms and susceptibility to gastric cancer. Gastric Cancer: Official J Int Gastric Cancer Association Japanese Gastric Cancer Association. 2011;14:317–21. 10.1007/s10120-011-0045-1. 10.1007/s10120-011-0045-1 21461653

[28] Theodoropoulos GE, et al. Polymorphisms of caspase 8 and caspase 9 gene and colorectal cancer susceptibility and prognosis. Int J Colorectal Dis. 2011;26:1113–8. 10.1007/s00384-011-1217-5. 21538054 10.1007/s00384-011-1217-5

[29] Elliott MR, et al. Nucleotides released by apoptotic cells act as a find-me signal to promote phagocytic clearance. Nature. 2009;461:282–6. 10.1038/nature08296. 19741708 10.1038/nature08296 PMC2851546

[30] Chekeni FB, et al. Pannexin 1 channels mediate ‘find-me’ signal release and membrane permeability during apoptosis. Nature. 2010;467:863–7. 10.1038/nature09413. 20944749 10.1038/nature09413 PMC3006164

[31] Lauber K, et al. Apoptotic cells induce migration of phagocytes via caspase-3-mediated release of a lipid attraction signal. Cell. 2003;113:717–30. 10.1016/s0092-8674(03)00422-7. 12809603 10.1016/s0092-8674(03)00422-7

[32] Gude DR, et al. Apoptosis induces expression of sphingosine kinase 1 to release sphingosine-1-phosphate as a come-and-get-me signal. FASEB Journal: Official Publication Federation Am Soc Experimental Biology. 2008;22:2629–38. 10.1096/fj.08-107169. 10.1096/fj.08-107169 PMC2493451 18362204

[33] Weigert A, Mora J, Sekar D, Syed S, Brüne B. Killing is not enough: how apoptosis hijacks Tumor-Associated macrophages to promote cancer progression. Adv Exp Med Biol. 2016;930:205–39. 10.1007/978-3-319-39406-0_9. 27558823 10.1007/978-3-319-39406-0_9

[34] Brecht K, et al. Macrophages programmed by apoptotic cells promote angiogenesis via prostaglandin E2. FASEB Journal: Official Publication Federation Am Soc Experimental Biology. 2011;25:2408–17. 10.1096/fj.10-179473. 10.1096/fj.10-179473 21450910

[35] Shin SA, Moon SY, Park D, Park JB, Lee CS. Apoptotic cell clearance in the tumor microenvironment: a potential cancer therapeutic target. Arch Pharm Res. 2019;42:658–71. 10.1007/s12272-019-01169-2. 31243646 10.1007/s12272-019-01169-2

[36] Zhang Y, et al. Single-cell analyses of renal cell cancers reveal insights into tumor microenvironment, cell of origin, and therapy response. Proc Natl Acad Sci U S A. 2021;118. 10.1073/pnas.2103240118. 10.1073/pnas.2103240118 PMC8214680 34099557

