# Supplementary figures and images for: Integrative SMR and single cell & spatial analysis reveals the spatial heterogeneity and prognostic value of CASP9-mediated apoptotic pathways in clear cell renal cell carcinoma
Source: Discov Oncol. 2025 Nov 7;16:2057. doi: 10.1007/s12672-025-03798-0 (PMC12595158; doi:10.1007/s12672-025-03798-0)

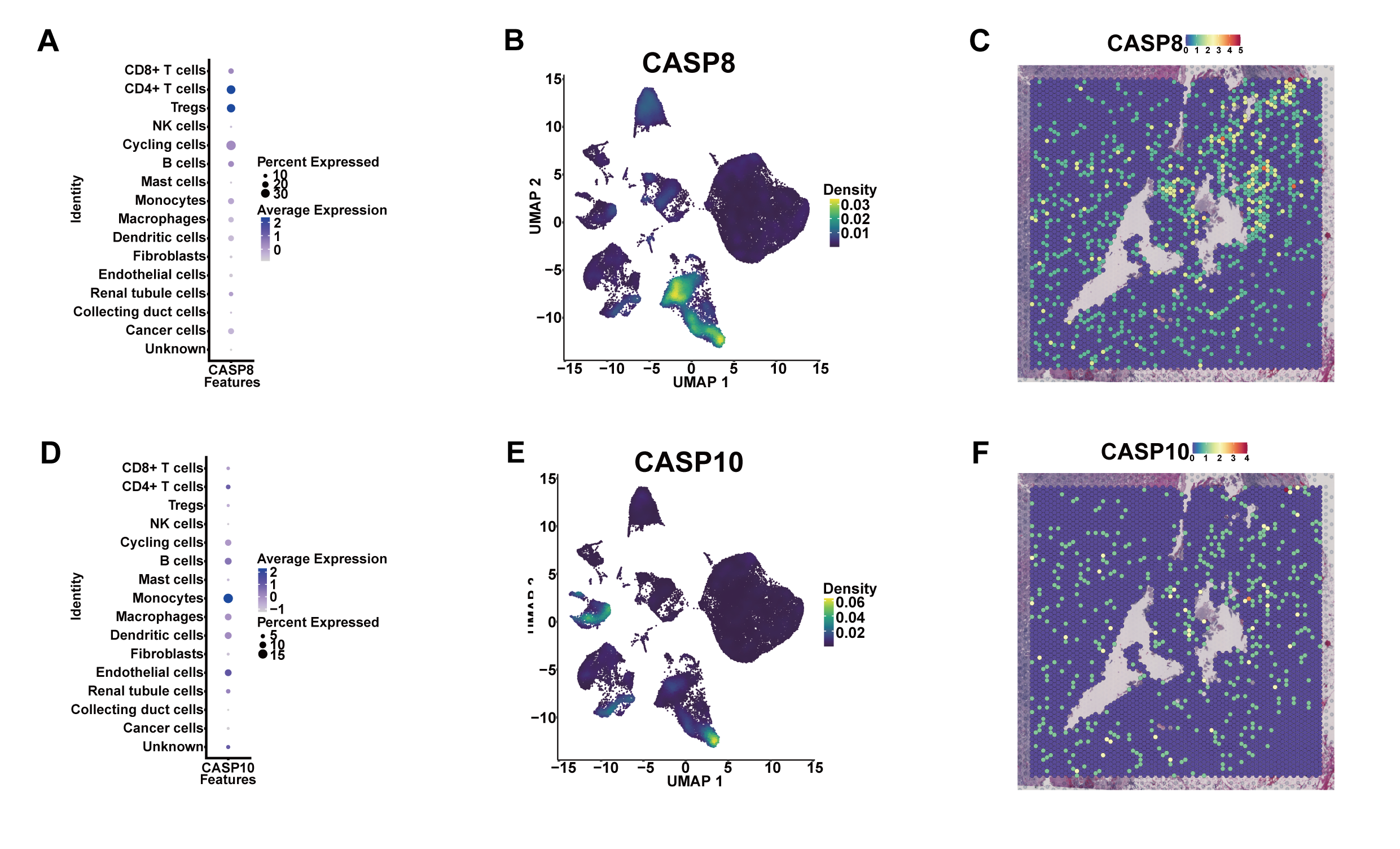

Supplement: Supplementary file 1 — Supplementary Material 1. [file 12672_2025_3798_MOESM1_ESM.tif]
